# ‘A Plentiful Crop of Cripples Made by All This Progress’: Disability, Artificial Limbs and Working-Class Mutualism in the South Wales Coalfield, 1890–1948^1^

**DOI:** 10.1093/shm/hku009

**Published:** 2014-04-07

**Authors:** Ben Curtis, Steven Thompson

**Keywords:** disability, artificial limbs, labour movement, mutualism, south Wales

## Abstract

Historians of orthopaedics, artificial limbs and disability have devoted a great deal of attention to children and soldiers but have neglected to give sufficient space in their studies to industrial workers, the other patient group that has been identified as crucial to the development of these areas. Furthermore, this attention has led to an imbalanced focus on charitable and philanthropic activities as the main means of assistance and the neglect of a significant part of the voluntary sphere, the labour movement. This article, focusing on industrial south Wales, examines the efforts of working-class organisations to provide artificial limbs and a range of other surgical appliances to workers and their family members in the late nineteenth and early twentieth centuries. It finds that a distinctive, labourist conception of disability existed which envisaged disabled workers as an important priority and one to which significant time, effort and resources were devoted.

In March 1919, Vernon Hartshorn, miners' agent of the Maesteg District of the South Wales Miners' Federation (SWMF) and Member of Parliament for the Ogmore constituency in Glamorgan, appeared before Justice Sankey's Coal Industry Commission. Hartshorn painted a vivid picture of the consequences of an industry continued without consideration for the well-being of the people of mining communities and with an eye to profits alone. Similar to many other witnesses who appeared on behalf of the miners' unions, Hartshorn drew the Commission's attention to the considerable perils that miners faced in the course of their daily labours. Each year roughly one in every six miners experienced a disabling injury that was sufficient to prevent them from working for seven days or more. He gave moving testimony of men and boys maimed, burnt and killed every working day:

In the mining industry the casualties are more like those of the battlefield than anything else. The only difference between the soldier and the miner is that the miner can never ask for an armistice. He cannot even treat for terms of surrender. The casualties go on every day.^2^

Delivered just a few months after the end of the First World War, the significance of Hartshorn's metaphor would not have been lost on anyone present. His rhetorical strategy was intended to elicit some of the massive amount of sympathy felt for the injured and maimed veterans of the Western Front on behalf of the mining population of Britain's coalfields. It was also perhaps intended to highlight the injustice felt by the people of those mining communities that the lot of injured miners had been neglected for so long and that it was being overshadowed in these post-war years by the overwhelming focus on military casualties. Attention to the ‘wounded soldiers of industry’, who were ‘always “in the trenches”’, in a ‘war that knows no end’, was to be a common theme in union rhetoric in the years that followed.^3^

The contrast in responses to military and industrial casualties was drawn again, with greater intensity, during the Second World War and was eloquently expressed by Bert Coombes, the ‘miner writer’ from Resolven in the Neath Valley:

On pay day at the average colliery you may see a long queue of men with bandaged arms, or heads, or swinging along on crutches. It has the appearance of a dressing station behind the battle front. There is no glory attached to this queue because they are the wounded of the industrial battle.^4^

The extent of such serious and frequently permanent injuries, on a literally industrial scale, prompted the development by the south Wales miners of a range of institutions and services to assist individuals who had become thus disabled. This article examines the efforts of working-class organisations in the south Wales coalfield, in which miners played a very prominent role, to provide artificial limbs and a range of other surgical appliances to workers and their family members in the late nineteenth and early twentieth centuries. What emerges from this study is that a distinctive, labourist conception of disability existed, which envisaged disabled workers as an important priority and one to which significant time, effort and resources were devoted.

## Industrial Workers and the Historiography of Disability

The neglect of industrial casualties, which Vernon Hartshorn described so movingly in 1919, is one that also extends to historiography. A number of authors have insisted that children, war veterans and industrial workers were the groups of the population that were crucial to the development of orthopaedics and to the changing experiences and understandings of disability in modern Britain.^5^ These groups suffered crippling injuries and impairment to a significant degree and they were the focus of the attention of surgeons and specialists in their attempts to develop modern orthopaedics and lessen the extent of crippling conditions within the population as a whole. Roger Cooter's observation in 1993 that children, soldiers and industrial workers are the patient groups of most importance to the making of modern medicine has been answered with a number of studies of children as patients—not least from his own pen—and particularly in the area of orthopaedics, and a veritable proliferation of studies of soldiers and their disabling conditions. A series of excellent studies have explored the nature and extent of crippling injuries caused by the wars of the nineteenth and twentieth centuries, the complexity of the responses to impairment, and the consequences for understandings and experiences of disability in the modern period.^6^ Industrial workers, however, continue to suffer the neglect of medical historians with little interest in working people, either as the objects of medical interventions or as agents in the making of modern medicine.^7^

Another feature of the historiographies of disability, orthopaedics and artificial limbs is the extent to which historians have focused their attention on the mixed economy of care and have assessed the relative importance of the state and charitable initiatives as the providers of assistance to crippled individuals. Time and again, historians have pointed to the overwhelming importance of philanthropy in British responses to crippling impairment, and its continued importance despite considerable state intervention during and following the First World War.^8^ Unfortunately, the importance attached to charitable activities is both a cause and a consequence of the neglect of other providers of care in the voluntary sphere, most notably the various organisations that constituted the British labour movement.^9^ Apart from a few isolated comments on the varied contributions of work-place collections, trade unions, and friendly societies in this context, historians have failed to give any real attention to working-class mutualism as a response to injury, limblessness and disability.^10^ Joanna Bourke, for example, claims that while disabled children were able to call on the sympathies of middle-class philanthropists, disabled adults were ‘socially invisible’. She also argues that the First World War had a transformative impact on the lives of all disabled people and turned them ‘from passive to active sufferers’.^11^ If we alter our perspective to include the labour movement, however, such disabled individuals come sharply into focus. Indeed, it might be argued that the needs of the disabled were assigned a high priority by certain working-class organisations and that the disabled were able to exercise a considerable amount of agency within and through such organisations, even in the period before the First World War.

## Industrial Injury in the South Wales Coalfield

Industrial south Wales provides a perfect case study for a consideration of industrial injury, impairment and working-class efforts to meet the needs of disabled individuals. The various industrial activities continued in the region from the late eighteenth century were particularly hazardous to the limbs and bodies of workers, to the extent that the *Morning Chronicle* correspondent who toured south Wales in 1850–51 commented that ‘I believe there are in Merthyr more men with wooden legs than are to be found in any town of the kingdom having four times its population’; the streets, he claimed, were ‘thronged with the maimed and the mutilated’.^12^ Such hazards increased with the massive development in the coal industry in the region from the 1870s onwards and, due to the geological nature of the coal measures, south Wales was the most dangerous coalfield in Britain. Large-scale disasters were more numerous, accident rates were higher, occupational disease was more common and, as a result, levels of injury and disablement were correspondingly higher than in other coalfields.^13^

In the years before the First World War, for example, a little over 1,000 ‘serious accidents’ occurred each year in south Wales and roughly 30,000 miners received injuries that caused disability lasting seven days or more.^14^ In each year during the 1920s, roughly 40,000 miners were victims of accidents that disabled them from working for seven days or more while the figure for Britain as a whole stood at about 200,000, and this did not include the numbers disabled by occupational disease. Every single working day, five miners were killed and 850 were injured in the mines of Britain.^15^ These figures compare with the 41,000 servicemen who lost an arm or a leg during the First World War and 272,000 men who experienced injuries to arms or legs that did not require amputation.^16^ The figures are not strictly comparable, of course, but give at least some indication of the relative scale of the casualties and perhaps help to make the miners' bitterness understandable.

Such high rates of accident and impairment created a large demand for artificial limbs and other surgical appliances, and there existed a variety of means by which these were provided to workers. Employers played at least some role, as the provision of artificial limbs formed part of a more general paternalism that was as much to do with the management of labour as it was the well-being of workers. Iron and, later, steel companies provided limbs to injured workers during the nineteenth century but such provision tended to be at the discretion of the employer or his manager and was conducted on a small scale only. Coal companies that came to prominence in the second half of the nineteenth century and the early years of the twentieth were even less generous in this regard and distinguished between ‘good’ and ‘bad’ workmen in their provision of assistance.^17^ While the miners' union insisted in the 1920s that the coalowners should be compelled to provide artificial limbs free of charge to injured miners, this was a demand that was never likely to be satisfied.^18^ The failure of employers to offer anything more than a minimal amount of assistance to injured workers and the relative absence of philanthropy from most colliery communities in the region, itself largely the result of the absence of any sizable middle class in coalfield towns and villages, left a space in which working-class self-help and mutualist efforts grew in importance as the nineteenth century progressed.

At the most basic level, injured individuals were succoured by work-place collections that would have allowed them to ease the problems caused by a reduced income or indeed to purchase an artificial limb. A slightly more sophisticated form of collections were the Art Union or ‘Prize’ draws, a type of lottery that involved the selling of tickets for a draw to win prizes donated by individuals, groups and companies in the community. In 1901, for example, a Prize Draw was held in the village of Llwydcoed, near Aberdare, in order to assist Jenkin Rees after he lost his arm in an accident at the Abergorki Drift.^19^ In a not dissimilar way, while friendly societies did not routinely provide the means by which members could procure or obtain artificial limbs, some branches made collections amongst their members in order to purchase artificial limbs for one of their number. ‘Brother’ John Morgan was presented with an artificial leg by the Merthyr Branch of the True Order of Ivorites in 1865, in a special meeting for the purpose replete with speeches, songs and a poem composed to the leg.^20^

## The Labour Movement and Medical Provision in the South Wales Coalfield

More significantly, certain aspects of the region's labour movement meant that the needs, interests and opinions of disabled workers were given a higher priority and greater status than in other industrial districts. First, in the South Wales Miners' Federation, founded in 1898, the region possessed a powerful trade union that was often in the vanguard of miners' trade unionism, and indeed the broader British labour movement as a whole, for much of the twentieth century.^21^ Membership passed 100,000 within a year of its inception, making it the largest miners' trade union in Britain by that time, and it was often the instigator of disputes or campaigns in the British coal industry, or else the most loyal region during such disputes, for most of its history. More relevantly, the Federation committed itself to taking an interest in all matters that affected the lives of its members and their families, and acted as advocate and defender in a broad range of areas. In particular, it found itself devoting so much time to the compensation cases of sick and injured members that one historian has claimed might be considered one of the Federation's most important functions.^22^

In addition, the region also possessed distinctive schemes of medical provision—usually described as medical aid societies—that were more robust, comprehensive and had a greater breadth of services than comparable workmen's medical schemes in other parts of Britain. These originated in the works' doctors schemes that were established in the late eighteenth and early nineteenth century at collieries and ironworks throughout the region, whereby employers appointed a surgeon to serve the medical needs of workers and their families and made compulsory deductions from wages to cover the doctors' salaries. In many instances, particularly in towns in northern Monmouthshire where iron and steel manufacture was carried on alongside coal production, workmen's committees were able to wrest some measure of control from their employers, institute set salaries for surgeons and utilise the surplus to develop a broader range of services for members and their families. The Tredegar Workmen's Medical Aid Society was to become the most famous of these schemes, due to its association with Aneurin Bevan, and, by the 1920s, it provided the services of five doctors, one surgeon, two pharmacists, a physiotherapist, a dentist and a district nurse, in addition to a range of additional services, to roughly 95 per cent of the town's population.^23^ Crucially, medical aid societies such as those found at Tredegar, Ebbw Vale and Blaenavon were important institutions in the provision of artificial limbs and other surgical appliances, and, from 1911, represented members' interests in relation to National Insurance.

The collective self-organisation of workers, therefore, was a central factor in the provision of artificial limbs within south Wales coalfield society. The specific organisational form that this took varied across the region, depending on local circumstances: the Federation Compensation Department noted in a letter to the Bute Merthyr Lodge in September 1934 that ‘[a]s you are aware there are several practices obtaining in the Coalfield’.^24^ Some Federation districts organised their own artificial limb funds, to which all their members belonged by virtue of their membership of the union: examples of this included the Afan District Artificial Limb Fund (established 1923), the Maesteg District Artificial Limb Fund (established 1928) and the Area No. 2 Artificial Limb Fund, which covered the Neath and Afan valleys and was in existence by February 1934, if not before.^25^ Not all districts of the Federation, however, were able to make this provision. In 1921, the Rhondda No. 1 District reluctantly resolved that the question of the provision of artificial limbs was too extensive for it to undertake itself and, presumably, individual lodges within the district made provision as best they could.^26^

In other instances, the miners were the largest occupational group within general geographically-based medical aid societies, such as the Tredegar Workmen's Medical Aid Society, and it is likely that the existence of powerful medical aid societies in such places rendered the need for Federation funds unnecessary in those particular areas. Broadly speaking, the trend was for the Federation schemes to be established later than the provision of the workmen's medical aid societies: there is little evidence of the former before the 1920s, whereas the latter were in some instances providing artificial limbs in the late nineteenth century. The work of these artificial limbs funds continued through the travails of the interwar years, both on their own and in conjunction with the state's National Insurance scheme, before their role became superseded by the advent of the National Health Service in 1948. Judging by the number of named individuals recorded in the minutes of these various organisations, it is clear that many hundreds, if not thousands, of people received artificial limbs and other orthopaedic and medical appliances, either for free or at a subsidised rate, as the direct consequence of the medical schemes established by workers.

**Fig. 1:**
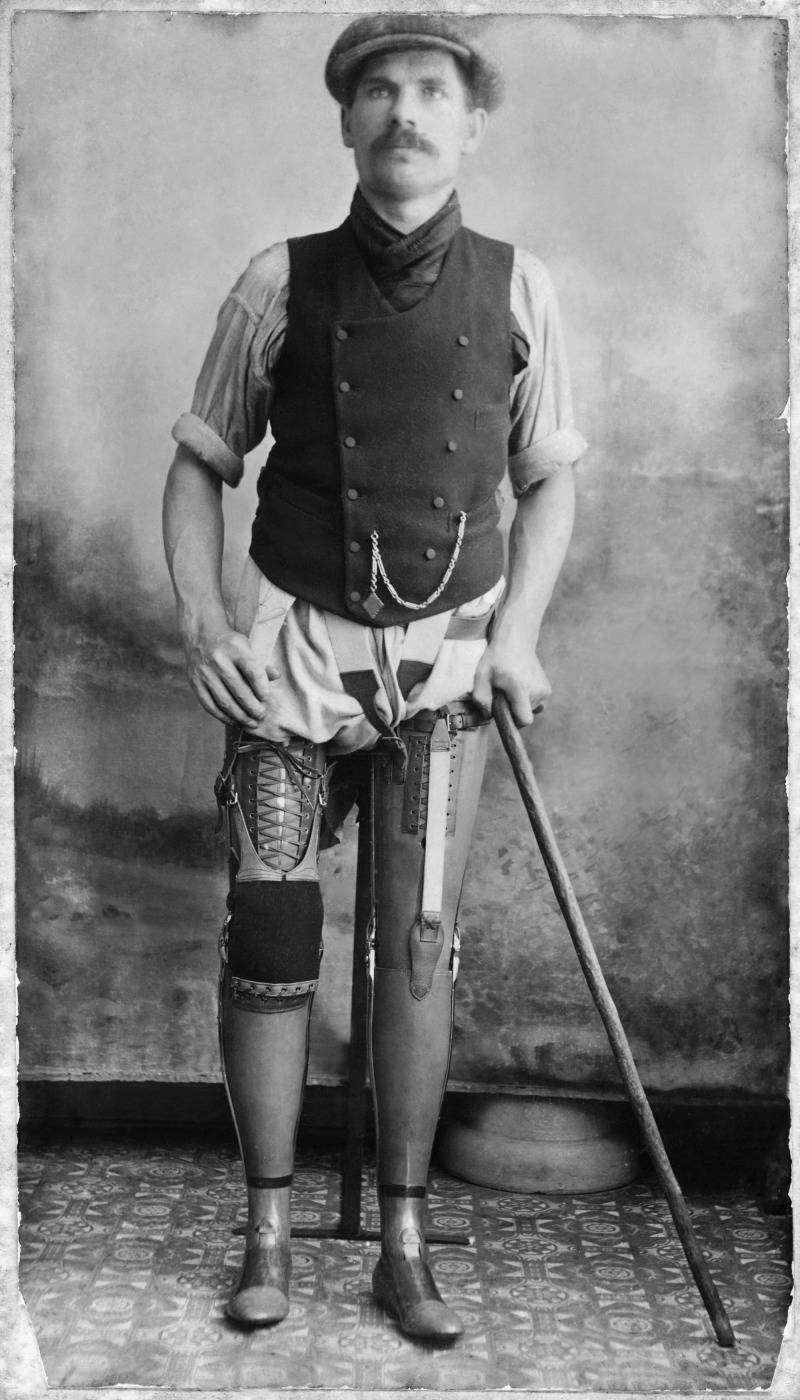
George Preece, a miner from Abercynon, injured in 1909 when a truck went over his legs. He is shown wearing technically sophisticated artificial legs (one full length, the other from the knee down) rather than simple wooden ‘peg legs’ – although, according to his granddaughter, he rarely wore them as they were not particularly comfortable. (Image courtesy of National Museum Wales)

One of the defining features of the artificial limbs fund schemes run by the south Wales miners was their inclusive and democratic character. Unlike the provision of limbs by charitable organisations and employers, which was inevitably an essentially philanthropic act dependent upon the providers' goodwill and prerogative, members of the workers' limb fund schemes were entitled to receive an artificial limb, as of right, on the terms specified by the scheme. Furthermore, the committees of both the artificial limb funds and the medical aid societies were selected by regular elections in which the respective memberships were able to pass judgement on the performance of those committees and thereby influence their decisions and policies. The distinction here, between a miners' system of ‘proletarian’ democratic entitlement and various instances of discretionary philanthropic or paternalistic provision elsewhere, was a significant one and speaks volumes about the popular culture within south Wales coalfield society at that time. Artificial limbs were not, as Joanna Bourke has claimed in relation to workers more generally, ‘a luxury’, but rather, in south Wales at least, something to be provided to those who needed them in a routine fashion.^27^

The specific rules defining the extent and nature of eligibility for receipt of artificial limbs varied within the different miners' limb fund schemes. In none of these schemes, though, is there any mention of a payment to be made by the limbs' recipients; full membership entitled the recipients to free provision of the appropriate artificial limb, not some sum of money towards its cost. With the various schemes run directly under the aegis of the Federation, the main criteria for eligibility were that the person in question was a full financial member of the Federation and that the accident which caused the individual to require the artificial limb occurred on colliery premises. Additionally, as these schemes were district-based, the individual in question generally needed to be employed within the appropriate district at the time of the accident. The rules of the Maesteg District Fund, for instance, make it clear that ‘The provision of this fund to apply only in cases of incapacity arising as a result of accidents sustained, or industrial diseases contracted, whilst employed in the Maesteg District, provided also that application is made whilst the workman is actually a member of the Maesteg District of Miners’.^28^ The terms of the Area No. 2 District fund were slightly more flexible in this respect. In March 1936, after consultation with the colliery lodges, it was decided ‘That no applications be considered from members who sustained accidents at Collieries outside this Area unless the applicant has been a contributor for at least five years’. In December 1943, in response to a motion from the Duffryn Rhondda Lodge to reduce the period of five years to three for the supply of a second artificial limb to individuals, the Area Committee meeting unanimously agreed to consider second applications upon their merit after a period of three years.^29^ One of the underlying characteristics of the various miners' artificial limbs fund schemes, therefore, was a humane flexibility and the desire to move beyond a strict interpretation of eligibility, albeit within the limits imposed by finite resources.

The question of eligibility and entitlement was slightly more complicated with respect to the various workmen's medical aid societies. In the case of the Tredegar Workmen's Medical Aid Society, members who paid ‘poundage’ contributions at a rate of 3d. per pound were entitled to either ‘Minor Benefits’ if they had paid continuously for three months, or ‘Major Benefits’ if they had paid continuously for a period of two years.^30^ An artificial (i.e. glass) eye was one of the items termed ‘Minor Benefits’, whereas an artificial leg was deemed a ‘Major Benefit’.^31^ At a special general meeting in July 1928, it was affirmed that the Tredegar Society's policy was for the dependent wives and children of members to be entitled to the free benefits ensuing from this. This meant that, unlike the provisions of the various Federation schemes, it was possible for women and children to receive an artificial limb (or, as was more likely, other surgical appliances) from this fund free of charge, providing the appropriate criteria were met. Such individuals received limbs as a result of their relationships with male members of the schemes, rather than as a result of their own particular needs, of course, but the provision was no less significant for that. The Society's resources were by no means unlimited, however; the same meeting decided that ‘only members who have contributed full poundage rates for the proper periods … shall be entitled to the benefits termed major benefits & that the second or duplicate artificial limbs be supplied only to members who are in actual employment’.^32^

In contrast, the general policy of the Ebbw Vale Workmen's Doctors' Fund (which by 1913 had changed its name to the Ebbw Vale Workmen's Medical Society) from March 1898 onwards was one of provision of grants of up to £4 per limb, rather than meeting the entire cost of artificial limbs. In March 1916, the Fund decided to pay for two-thirds of the cost of artificial limbs for members, up to a maximum of £8 for legs and £5 6s. 8d. for arms and hands. These increased rates of grants were considered retrospective as of August 1917, so as to remove any inconsistencies.^33^ The policy of the Ebbw Vale Society, first stated in December 1898, was that these artificial limb grants were to be for injuries newly sustained, rather than for injuries incurred prior to membership.^34^ A proposal to extend this policy to include grants for limbs lost prior to membership for payees of ten years' standing was defeated in November 1921 and the status quo seems to have remained thereafter.^35^ Clearly, financial reality placed limits on the generosity of provision.

Notwithstanding the fact that the various rules of the miners' artificial limb fund schemes did not always permit the provision of prostheses to members, one of the most notable aspects in the way that they were run was that occasionally, where circumstances necessitated, the various managing committees were able to use their discretion to extend provision to individuals who would otherwise have been ineligible; such instances show that the schemes were not always run according to strict rules of eligibility or on an actuarial basis and that the need of an individual alone could be the basis on which provision was made. Several examples from the Federation Area No. 2 Artificial Limbs Fund illustrate this point clearly. In July 1934, the Area committee considered the case of a member of Duffryn Rhondda Lodge and decided ‘in view of the circumstances it was agreed that a grant of £5 be made towards the cost of a new Limb’.^36^ In November 1942, the Garth Merthyr delegate on the Area committee appealed on behalf of a member of the lodge who required orthopaedic boots. The meeting ‘decided that the case did not come within the scheme but agreed to a grant of £3.3.0 which was half the cost of the Surgical Boots’.^37^

The same ethos was apparent in the workings of the various workmen's medical aid societies. In September 1936, for instance, the Tredegar Society's General Committee resolved that ‘a member whilst disabled, is entitled to [the] full benefits of the Society'.^38^ Similarly, in June 1938 the Society received an application from an individual for a new wooden ‘peg leg’. This miner had formerly worked at Graham's colliery, near Tredegar, which had closed in the early 1920s and whose workforce had been contributors to the Society. The Society had previously supplied this individual with two legs out of the proceeds of a benefit football match and the Committee ‘finally agreed he be supplied with a new peg leg free of cost’.^39^ Responsiveness to members' complaints was another feature. When, in November 1933, the Tredegar Society's Economy Sub-Committee proposed that wearers pay for 50 per cent of the cost of repairs to artificial limbs, it received a petition of protest from five wearers of artificial limbs. Dissatisfaction with this aspect of the Society's policy persisted for over a year, before, in response to these complaints, the Society decided in August 1935 to rescind charges to members for repairs.^40^

Workers were not the sole beneficiaries of the benevolent outlook of these societies. In March 1938, a woman applied to the Tredegar Workmen's Medical Aid Society for repairs to her artificial limb, which she had worn for the previous seventeen years. However, on being advised by the manufacturers as to the age and poor condition of the prosthesis, it was decided to pay £20 to supply her with a new artificial limb instead.^41^ Similarly, in March 1943, a woman appealed to the Ebbw Vale Society for assistance towards the price of her artificial leg, to which the Society's Hospital Committee responded by agreeing to provide a grant for two-thirds of the cost.^42^ Orthopaedic equipment was also provided to assist with treating congenital disabilities of the dependants of members. In November 1900, for example, in response to a member's request for assistance, the committee agreed to pay for the ‘cost of [a] steel arrangement to support [the] limbs of his daughter, 6 years of age and a cripple’ providing a further grant four years later to the same individual ‘to defray [the] cost of leg supports for his little girl’. In July 1904, it was resolved to pay to another member 12s. 9d., this sum being ‘the cost of Leg supports for his little son’.^43^

These examples do not mean, of course, that artificial limbs and other surgical appliances were provided by these funds without any consideration of the funds' rules or finances. Individuals' applications *were* occasionally refused. In January 1938, the Tredegar Society's General Committee ‘reaffirm[ed] our previous resolution, that no second artificial limb shall be supplied to a member who is not working’.^44^ Likewise, in March 1944 the Federation's Area No. 2 District Committee turned down the application by a former Duffryn Rhondda miner on the grounds that ‘This man had been employed at the R.O.F. [Royal Ordnance Factory] for several months and … he could not now be regarded as a member of the Federation’.^45^ The following year, in response to two instances of individual miners placing orders for artificial limbs without having first sought the approval of the union, the Area No. 2 Committee ‘agreed that in future when members ordered Limbs direct and not through the Area that payment should not be granted’.^46^

## Miners' Medical Aid Societies and Disability in the South Wales Coalfield

The various medical aid schemes run by workers in south Wales provided an array of surgical appliances for members, a reflection of the fact that work in the coal industry produced a wide range of permanent and semi-permanent disabling conditions and injuries amongst its employees. This is a significant point, inasmuch as the historiography of disablement and prostheses has tended to concentrate on amputations and artificial limbs, to the exclusion of other (more commonplace) conditions and orthopaedic devices.^47^ The rules of the Maesteg District Artificial Limb Fund, for example, stated that its purpose was to supply artificial legs, arms, feet, hands, fingers, eyes and teeth, as well as bath chairs, orthopaedic boots, surgical belts, spinal corsets and spectacles.^48^ Naturally, the range of appliances was not static and altered over time in response to a variety of factors, including medical technological developments, the requirements of the funds' members, and the financial position of the funds themselves. Nevertheless, the long-term trend seems to have been towards an expansion in the breadth of provision. In 1912, for example, the Tredegar Workmen's Medical Aid Society decided to establish a fund ‘[t]o procure Spinal Carriages & other appliances for aged and disabled workmen and their families’. By 1924, this had become a much more developed and structured scheme providing a very similar range of appliances to those supplied by the Maesteg District Artificial Limb Fund listed above, sub-classified into ‘Minor Benefits’ and ‘Major Benefits’.^49^

Artificial limbs were the most prominent and expensive items provided, although they only represented a minority of the number of surgical and orthopaedic appliances that were supplied. Crutches were a fairly commonplace item. In some instances, these were loaned to an individual on a temporary basis while he or she recovered from an injury; in other cases—such as those of a person suffering from rheumatism, for example—the provision of crutches was deemed to be permanent. Orthopaedic boots (generally referred to as ‘surgical boots’) were the items most often supplied by the medical aid societies. Demand for them was noticeably lower amongst members of the Federation artificial limbs funds, a reflection of the fact that the former organisations tended to cater for families and a wider section of the community than just the mineworkers themselves. In 1898, for example, Edwin Walters from Manmoel was granted the sum of £5 18s. 6d. by the Ebbw Vale Fund for ‘boots for his 2 children afflicted with Club feet’.^50^ In the dire economic circumstances of interwar industrial south Wales, the provision of such specialist orthopaedic footwear by the medical aid societies would have been gratefully received by workers' families. On occasion, the provision of surgical boots shaded into a more comprehensive orthopaedic treatment. In February 1928, for example, the Tredegar Society agreed to pay for ‘boots & irons’ for a child.^51^ Similarly, there are also a few instances of provision of a spinal jacket to correct spinal curvature.^52^

The extremely physical and arduous nature of coal-mining work meant that hernias and other serious strains were commonplace for miners; the rigours of the general business of daily life in coalfield communities meant that many women were also afflicted, as the records of the medical aid societies testify. Although rarely fatal, hernias and other such injuries would have been a source of continual pain and discomfort that impacted materially on a miner's income, given that most wages in the industry at that time were paid on a piecework basis according to the individual's output. Consequently, perhaps the surgical appliances most frequently supplied in the south Wales coalfield in this period were the various surgical trusses, abdominal belts and spinal corsets which were provided to help alleviate the symptoms of these conditions. In broad quantitative terms, approximately 25 to 50 per cent of all applications made to the Area No. 2 Artificial Limbs Fund in each year between 1934 and 1948 were for trusses. Similarly, there are 165 mineworkers named in the Dowlais Iron Company employees' ‘Truss and Wooden Leg Register’ for the period 1891–1902, the vast majority of whom required surgical trusses of various types.^53^ Many of these individuals apparently wore trusses for the duration of the 1891–1902 period covered by the extant records and it is entirely possible that such injuries would have been very long-term, if not permanent. The very large number of applications for surgical trusses and similar orthopaedic appliances is eloquent testimony to the endemic nature of chronic, debilitating conditions in coalfield communities.

At the other end of the scale, the various south Wales miners' artificial limb funds also occasionally provided much more specialist equipment for their disabled members. The wheelchair (or ‘invalid chair’, as it tended to be referred to in the early twentieth century) is one such example. Medical aid societies and artificial limb funds used their own resources, obtained via membership subscriptions, to purchase (or assist with the purchase of) wheelchairs for their members where necessary. The Tredegar Workmen's Medical Aid Society operated a small pool of chairs and spinal carriages.^54^ The general policy appears to have been one of loaning these out to disabled or elderly and infirm members on demand rather than giving them out permanently, although there do seem to have been occasional exceptions in this respect.^55^ In December 1922, the Finance Committee of the Tredegar Society resolved that non-members could hire a bath chair from the Society for a fee of five shillings per week.^56^ Commencing with the acquisition of a spinal carriage in 1912, the Society obtained and maintained several kinds of chairs for disabled members: bath chairs (an early type of wheelchair), folding chairs (which were collapsible and thus easier to transport) and Bailey chairs (a static, adjustable seat with supports for legs).^57^

There are other examples from other organisations too. In December 1939, for instance, the Federation's Area No. 2 Artificial Limb Fund agreed to provide money to assist with the purchase of a ‘Motor Chair’ for an individual; it also provided a grant of £25 to a member of Empire Lodge towards ‘the purchase of a mechanically propelled chair’ in December 1948.^58^ The provision of artificial eyes suggests the important place of aesthetic, as well as more functional, considerations in the provision of these workers' organisations. The intention was clearly to do more for the member than merely allow him to seek and obtain further employment; in these cases, the member's quality of life and sense of self-esteem was a motivating factor.^59^ The same is also true of the hearing amplifiers that were occasionally provided to assist individuals who were deaf. Such devices could be very expensive: in September 1945, for example, the South Wales NUM Area No. 2 Artificial Limb Fund agreed to purchase a device for one of its members in the Rhigos Lodge, at a cost of £23 2s. 0d.^60^ Both practical and aesthetic considerations played a part in the decisions taken by these medical aid societies and artificial limbs fund schemes.

Nevertheless, the provision of prostheses was the central rationale of the funds and artificial limbs were clearly the most expensive and most prominent of the appliances supplied to members. In the years immediately following the First World War, at a time when the average weekly wage for a skilled worker was £2, a wooden artificial leg could cost over £22.^61^ The ‘usual type’ of artificial leg supplied by the Tredegar Society cost £16 10s. 0d. in 1934, whereas a ‘Metal Leg’ cost £18 0s. 0d.^62^ Although not directly comparable, these lesser figures indicate that the Society was unable to spend as much per prosthesis as the government, the main purchaser of artificial limbs in Britain at that time. Even so, the provision of artificial limbs by these various miners' organisations did entail considerable expense. Between 1916 and 1948, for instance, the Tredegar Workmen's Medical Aid Society spent over £61,809 6s. 8½d. (an average of approximately £2,060 a year) on surgical appliances—a truly remarkable sum, given the extent of the economic depression in industrial south Wales.^63^

## Quality, Suitability and Value for Money

Given the scale of the costs involved and the finite nature of the financial resources at their disposal, a recurrent concern of the various miners' artificial limbs fund schemes was to try to obtain the maximum possible value for money for any prostheses purchased. The rules of the Area No. 2 Artificial Limbs Fund of the Miners' Federation, for example, stipulated that ‘It shall be the duty of the Area Officials to get quotations from as many firms as possible and secure a guarantee for each limb or eye supplied’.^64^ A similar imperative was discernible in the deliberations of the Tredegar Workmen's Medical Aid Society: by later 1935 its General Committee had become dissatisfied with the cost of the service provided by M. Masters and Sons Ltd, the main manufacturer of artificial limbs used by the Society between 1923 and 1936. Indeed, the relatively high charges made by Masters seem to have been an important factor in the switch to C. A. Blatchford and Sons Ltd in 1936–37 as its preferred supplier of artificial limbs.^65^ In January 1938, the Society's General Committee introduced a policy of a three-month trial period for artificial limbs to ensure the wearers' satisfaction with them before payment, terms to which Blatchfords indicated that they were agreeable.^66^

The quality of construction of artificial limbs was another important consideration. In the early 1920s, the Tredegar Society's preferred supplier of prostheses was the Cardiff branch of J. J. Stubbs and Son Ltd. Under the heading ‘A Boon to the Lame’, a 1920 advertisement for Stubbs' artificial limbs claimed that ‘The motions and actions are as near like a natural foot as possible, no springs, bolts, etc., to get out of order. The yielding and elastic qualities of rubber supply requisite motion; avoid all jars to the stump when walking; absolutely noiseless’.^67^ In late 1922, however, the Society's secretary was obliged to write to Stubbs on several occasions regarding the quality of their artificial limbs. In May 1923, the Finance Committee agreed to authorise Masters and Sons Ltd to provide three prostheses on a ‘trial’ basis; Masters subsequently became the preferred supplier of artificial limbs to the Tredegar Workmen's Medical Aid Society for the next thirteen years or so.^68^

Significantly, the design and technical construction of prostheses supplied by the south Wales miners' artificial limbs fund schemes varied over time, with the tendency seeming to have been towards improvements in form and function.^69^ These miners' schemes differentiated between ‘artificial legs’ and much less technologically sophisticated ‘peg legs’, with the overwhelming trend being towards provision of the former rather than the latter. In contrast, employers were generally only willing to supply peg legs to injured workers.^70^ The one type of prosthesis was considerably more expensive than the other: in 1897, for instance, the Ebbw Vale Committee noted that the cost of ‘a foot and socket leg’ was about £6 6s. 0d., whereas peg legs cost about £1 1s. 0d. each.^71^ As Guyatt notes, the main technological trend in construction of artificial legs in the interwar period was away from wood and towards metal as the preferred material.^72^ This shift is also discernible in the Tredegar Workmen's Medical Aid Society records: in April 1934, for instance, the General Committee agreed that one woman be supplied with a ‘Metal Leg at cost of £18 as against the usual type of £16.10.0.’ Similarly, specific references to artificial legs made of steel and aluminium first appear in the minutes in 1936 and 1937 respectively.^73^ By way of contrast, veterans of the First World War were receiving aluminium-alloy artificial legs from the early 1920s onwards, paid for by the Ministry of Pensions—a reflection of the far greater resources at the disposal of the government compared to the various miners' artificial limbs funds in the south Wales coalfield.^74^ On the other hand, Joanna Bourke makes the point that, as late as the 1930s, ‘unless a disabled person had access to charitable or private funds, he or she was not likely to be fitted with any artificial limb more sophisticated than a peg leg’ and asserts that this did not change until after the Second World War; workers in south Wales, in contrast, seem to have had access to metal limbs slightly earlier as a result of the efforts of their organisations.^75^

From the wearer's perspective, an equally significant factor was that an artificial limb was suitable and fitted correctly. In order to ensure this, it was generally necessary for the person to travel to the artificial limb manufacturer's workshop for measurement and for the limb to be fitted. The miners' medical aid societies sometimes paid the train fares for these journeys, where circumstances necessitated. In February 1906, for example, the Ebbw Vale Workmen's Doctors' Fund agreed that a member from Waunlwyd ‘be allowed his trainfares to & fro[m] Bristol, for the purpose of procuring an artificial leg’. The leg cost £3 3s. 0d. and was subsequently presented to him for free.^76^ Similarly, in the case of a boy who required prostheses, the Ebbw Vale Society's Hospital Committee resolved in September 1917 that ‘[t]he Secretary was instructed to get the boy … to Cardiff to see Dr Lynn Thomas as to suggested artificial arms’.^77^ Perhaps mindful of these travel costs for its members, in May 1921 the Tredegar Workmen's Medical Aid Society was able to persuade Mr Stubbs, of Stubbs and Son Ltd, ‘to come to Tredegar to measure persons for new artificial limbs at reduced prices’, as well as making the necessary arrangements for his visit. Thereafter, the policy of the Society was ‘to pay train fare only for persons going to Cardiff in respect to mis-fits with their artificial limbs’.^78^ Julie Anderson has noted the varied experiences of ex-servicemen and injured workers in terms of the continued care and support offered to the former but denied to the latter by orthopaedic surgeons and artificial limb makers in the 1930s; workers in south Wales, it is clear, as a result of the efforts of working-class organisations, had similar experiences to ex-servicemen rather than other workers in the long-term support they received in relation to their artificial limbs.^79^

It is illustrative of the Tredegar Society's underlying ethos of provision that, where circumstances allowed, on several occasions in the later 1930s, the Society sought the opinion of wearers as to their preference for an artificial limb. One individual said that he would like an artificial leg of all-leather construction; in other instances the Society's General Committee decided that the person in question ‘be provided with a new limb of his own choice’.^80^ Inevitably, it was sometimes the case that an artificial limb was either unsuitable, defective in some way, or incorrectly fitted. In January 1938, the General Committee resolved ‘[t]hat members who should accept limbs from the Society, do so on the condition, that after receiving the limb, if we do not receive a report if such limb is right or wrong within three months we refuse any application for repairs or a new limb’.^81^ Where a member duly informed the Tredegar Workmen's Medical Aid Society of an incorrectly-fitting or defective prosthesis, the Society's General Committee seems to have been quite zealous in ensuring that the grievance received adequate redress. To take one example: a member of the Society had been provided with a Masters artificial leg, which had proved to be unsuitable. In December 1937, having been unable to get Masters to meet a deputation to discuss this complaint, the committee resolved that the person be supplied with a Stubbs artificial leg instead. This replacement also proved unsatisfactory, despite Stubbs adjusting the limb twice to meet his specifications. In March 1938 the committee invited the individual to appear before them; upon examining the limb themselves, the committee's lay experts expressed the view that the limb itself was a misfit. Committee representatives subsequently accompanied the individual to the Stubbs workshop in Cardiff, where a further fitting—lasting three hours—took place. In July 1938, the wearer complained again that the limb was still unsatisfactory, prompting the General Committee to arrange for a meeting with the branch manager of the Stubbs workshop to resolve the issue. The following month, it was reported that the branch manager had conceded to the wearer that ‘he certainly was justified in his complaints’ and had agreed to make the necessary adjustments to it.^82^ The Company, perhaps, was keen not to lose as valued a customer as this workmen's medical scheme.

Repairs and replacements for damaged artificial limbs is one area where several of the south Wales miners' artificial limbs fund schemes went beyond the basics of provision to give an enhanced service to their members. The Area No. 2 Artificial Limb Fund of the Miners' Federation frequently authorised repair or replacement of damaged prostheses and surgical appliances. The emphasis was on repair where possible, replacement where necessary. The fund's rules, revised in 1936 following circulation to the lodges for discussion and approval, stated ‘That second applications for Artificial Limbs be not considered within the period of guarantee (nominally five years) except in case of severe accident to the Limb’, but ‘That the cost of repairs to Limbs be granted within the period of guarantee if necessary for the purpose of prolonging the life of the Limb. Particulars of such repairs to be sent with the applications’ and ‘That second and subsequent applications for repairs be considered on merit’.^83^

The Tredegar Workmen's Medical Aid Society operated on a similarly comprehensive basis, to the great benefit of some of its prosthesis-wearing members. Several individuals required multiple repairs to their artificial limbs: one particular person, for example, had his artificial leg repaired seven times and adjusted on a further occasion between 1927 and 1936, all of which were paid for by the Society.^84^ In certain circumstances, the General Committee deemed that replacement of a damaged artificial leg would be more cost-effective overall than repairing it: this occurred in June 1938, for example, following receipt of a quotation from Blatchfords Ltd of £11 5s. 0d. for repairs to a member's prosthesis.^85^ It should be noted, however, that not all of the artificial limbs fund schemes operated on this basis: the rules of the Maesteg District Artificial Limbs Fund stated unambiguously that ‘[t]he scheme does not provide for renewals’, while the general policy of the Ebbw Vale Workmen's Medical Society policy was to not to accede to members' requests for the Society to pay for repairs to limbs or to provide further grants towards the cost of replacement limbs—although there were occasional exceptions to this.^86^ Furthermore, even where the funds did pay for repairs and replacements of artificial limbs and surgical appliances, this provision was dependent upon continued membership of the scheme; it was occasionally the case that individuals' requests for assistance were not acceded to as they did not meet this criterion.

The question of repairs also exemplifies the Society's commitment towards meeting the needs of its disabled members. This is illustrated clearly by a series of interconnected examples from the mid- to late 1930s. Two themes emerge from these: the desire of the General Committee to get members' limbs repaired as rapidly and cost-effectively as possible; and the lay expertise of several of the committee members and officers in the question of artificial limbs. In October 1935, concerned at the high cost of repairs to artificial limbs charged by Masters and Sons, the General Committee resolved that the limbs be returned from that company and inspected by a sub-committee before these repairs be proceeded with. The following month, the sub-committee reported its deliberations, after which the General Committee itself inspected the prostheses in question and resolved that the sub-committee meet with the representative from Masters and Sons and gave it ‘plenary powers to arrange for repairs, also to try and arrange with him to supply us with a catalogue of prices on all repairs generally, as a guide for the future’. Following this meeting with Masters and Sons, the sub-committee was able to negotiate a discount of ten shillings on the repair bill for each of the four prostheses in question. The General Committee resolved that this work be carried out ‘but that when any other limb required repair, the catalogue of prices (which is being forwarded), be checked, and decided upon by Committee. In the case of a new limb to be ordered, first competitive prices be obtained, and a guarantee asked for, and instructions issued by the Firm who supply the limb, as to use and adjustments to that limb’.^87^

This question of the high cost of Masters and Sons' repairs re-emerged in April 1936. One committee member ‘raised [the] question of obtaining competitive prices for repairs, but [the] Secretary stated this would mean the limb being put to each Firm in turn, the member having to wait a long period on this system for return of repaired limb’. The sub-committee met the Masters and Sons representative again but was unable to negotiate a further discount. A few months later, though, the secretary had been able to ascertain that Blatchfords were able to repair artificial limbs at a price 50 per cent below that of Masters and Sons; this was an important factor in the switch to this company as the preferred manufacturer used by the Society. On several other occasions too, certain committee members examined defective or faulty artificial limbs that had been supplied to Society members, with the (generally successfully obtained) objective of getting a free replacement or repair of the prosthesis in question.^88^ Interestingly, in the late 1930s, one of the General Committee's artificial limbs lay experts was himself a disabled individual who wore an artificial leg.^89^ Elected onto the committee in April 1936 as one of the two representatives from Pochin No. 1 colliery, he subsequently played a prominent role in the small sub-committee that examined damaged or faulty artificial limbs and negotiated with the manufacturers regarding replacements or repairs to these.^90^

## Disabled Miners, Artificial Limbs and the Return to the Workplace

The significant role of the Pochin No. 1 representative on the committee raises the question of the extent to which disabled individuals who wore artificial limbs were able to re-enter the workplace. In certain circumstances, there sometimes existed the possibility of suitable light employment outside of the coal industry: one individual from Sirhowy who wore an artificial leg, for example, was able to obtain work as a billiard marker in the local miners' institute.^91^ Generally speaking, though, the evidence certainly indicates that many individuals with artificial limbs were able to continue to work in the coal industry; what is less clear is in what jobs they were employed. Whilst the statistical data do not exist so as to be able to quantify precisely, the aggregate weight of case studies and anecdotal evidence regarding individuals who lost a limb suggests that where possible they were generally offered ‘light work’, either on the colliery surface or elsewhere below ground, away from the arduous physical business of cutting coal at the coalface. Working in the colliery lamp room was fairly commonplace;^92^ other options included working as an engine driver or on the colliery ventilation fans.^93^ The provision of the artificial limb was a necessary factor in this person's work performance—albeit almost certainly at a lower wage than before the disabling injury occurred. In this respect, it is clear that the many thousands of pounds' worth of prostheses and other surgical appliances funded by the various miners' artificial limb fund schemes from the 1890s through to the 1940s played an important role in helping the numerous victims of disabling industrial injury in south Wales coalfield society to have a greater level of income and independent living than would otherwise have been the case.

## Conclusion

While the attention within the historiography of orthopaedics and artificial limbs has been paid to soldiers and children, attention devoted to workers can further broaden our understanding of these important issues and offer a distinct contribution to the field. What is evident in these working-class organisations in this particular part of Britain is a distinctive, labourist conception of disability, need and entitlement that differed quite markedly from state and charitable conceptions. It placed the disabled individual in a much more favourable position than other civilians, deserving of assistance as a right, and conferred eligibility to a considerable amount of care and on-going support. Indeed, in terms of the large sums of money raised and expended, the dogged and continuous representations made to artificial limb companies, and the campaigning work of the Miners' Federation to improve workmen's compensation legislation, the labour movement placed the disabled worker at the heart of its efforts and campaigns, and this clearly stands in contrast to the far more limited, discretionary character of provision by other sectors of the voluntary sphere.

It should also be noted that this commitment to the well-being of disabled individuals was not inevitable but came about as a result of careful, conscious decisions that placed the disabled at the heart of the labour movement's activities. This is clearly demonstrated by a comparison with the Railway Brotherhoods in America where disability was a divisive issue as injured railwaymen were criticised by their brethren who attributed disability to incompetence and used it as a means to limit the amount of assistance given to such individuals.^94^ In south Wales, injured miners, metal-workers and even family members were taken up as part of the everyday work of the labour movement, or else such individuals pursued their interests through that labour movement, and a distinctive conception of disability was fostered. This was reflected in the breadth of provision made by proletarian organisations, in terms of the range of surgical appliances provided and the provision made to members' dependants, the relatively generous terms on which such provision was made, the continued support to those individuals assisted after provision had been made, and the commitment to less functional forms of provision, such as artificial eyes and hearing aids, that were nevertheless intended to improve the quality of individuals' lives.

## Funding

This work was supported by the Wellcome Trust [grant number 095948/Z/11/Z].

## References

[1] The comment in the title, ‘A plentiful crop of cripples made by all this progress’, was made by two socialists who toured south Wales in the 1880s. In their analysis, industrial capitalism sacrificed the flesh and limbs of workers in the pursuit of profit: ‘Shorn of a leg or an arm, they were painfully fulfilling their part in “progress”’, they claimed; *Commonweal*, 27 August 1887, article reproduced in Ken John, ‘Sam Mainwaring and the Autonomist Tradition’, *Llafur*, 1986, 4, 65.

[2] Coal Industry Commission. Vol. I. Reports and Minutes of Evidence on the first stage of the inquiry, [Cmd. 359], 1919, xi, 2–3.

[3] For other examples, see *The Colliery Workers' Magazine*, January, 1923, 1, 5; and January, 1924, 2, 23; *Further Facts from the Coal Commission, compiled by R. Page Arnot* (London: Frank Hodges, 1919), 22; Arthur Horner, *Coal Crisis: The Miners' Reply* (London: Daily Worker League, 1944), 16.

[4] Coombes B. L., Those Clouded Hills (London: Cobbett, 1944), 54

[5] See, for example, Roger Cooter, ‘The Disabled Body’, in Roger Cooter and John Pickstone, eds, *Companion to Medicine in the Twentieth Century* (London: Routledge, 2003), 367–83; Julie Anderson, *War, Disability and Rehabilitation in Britain: ‘Soul of a Nation’* (Manchester: Manchester University Press, 2011), 7, 14–41.

[6] Cooter Roger, Surgery and Society in Peace and War: Orthopaedics and the Organization of Modern Medicine, 1880–1948 (Basingstoke: Macmillan, 1993), 7. On children, see Roger Cooter, ed., *In the Name of the Child: Health and Welfare, 1880–1940* (London: Routledge, 1992); Harry Hendricks, *Children: New Perspectives—Child Welfare in England, 1872–1989* (London: Routledge, 1994); on war veterans and disability, see Lisa Herschbach, ‘Prosthetic Reconstructions: Making the Industry, Re-Making the Body, Modelling the Nation’, *History Workshop Journal*, 1997, 44, 22–57; Mary Guyatt, ‘Better Legs: Artificial Limbs for British Veterans of the First World War’, *Journal of Design History*, 2001, 14, 307–25; Deborah Cohen, *The War Come Home: Disabled Veterans in Britain and Germany, 1914–1939* (Berkeley: University of California Press, 2001); Joanna Bourke, *Dismembering the Male: Men's Bodies, Britain and the Great War* (London: Reaktion, 1996); Jeffrey S. Reznick, *Healing the Nation: Soldiers and the Culture of Caregiving in Britain during the Great War* (Manchester: Manchester University Press, 2004); Anderson, *War, Disability and Rehabilitation*.

[7] Historians of American workers have done better in this regard; see, for example, John Williams-Searle, ‘Cold Charity: Manhood, Brotherhood, and the Transformation of Disability, 1870–1900’, in Paul K. Longmore and Lauri Umansky, eds, *The New Disability History: American Perspectives* (New York, 2001), 157–86; Edward Steven Slavishak, *Bodies of Work: Civic Display and Labor in Industrial Pittsburgh* (Durham, 2008).

[8] For examples of the focus on the mixed economy of care in this context, see Anderson, *War, Disability and Rehabilitation*, *passim*; Cohen, *The War Come Home*.

[9] For exceptions, see Anne Borsay, ‘“Fit to Work”: Representing Rehabilitation on the South Wales Coalfield during the Second World War’, in Anne Borsay (ed.), *Medicine in Wales c.1800–2000: Public Service or Private Commodity?* (Cardiff: University of Wales Press, 2003), 128–53; Kim Howells, ‘Victimization, Accidents and Disease’, in David Smith (ed.), *A People and a Proletariat: Essays in the History of Wales 1780–1980* (London: Pluto Press, 1980), 181–98.

[10] For such comments, see Bourke, *Dismembering the Male*, 47–9; Anderson, *War, Disability and Rehabilitation*, 23–5; and, more substantially, Cooter, *Surgery and Society in Peace and War*, chs 7 and 9.

[11] Bourke, *Dismembering the Male*, 39, 44.

[12] Ginswick Jules (ed.), Labour and the Poor in England and Wales 1849–1851: The Letters to The Morning Chronicle. Vol. III The Mining and Manufacturing Districts of South Wales and North Wales (London: F. Cass, 1983), 49

[13] Benson John, British Coalminers in the Nineteenth Century: A Social History (Dublin: Gill and Macmillan, 1980), 37–43; Roy Church, *The History of the British Coal Industry. Volume 3: 1830–1913: Victorian Pre-eminence* (Oxford: Clarendon, 1986), 582–96.

[14] Jones Dot, ‘Workmen's Compensation and the South Wales Miner, 1898–1914’, Bulletin of the Board of Celtic Studies, 1980, 29, 135

[15] The Colliery Workers' Magazine, November, 1923, 1, 268; May, 1924, 2, 114–15; and September, 1926, 4, 190.

[16] Bourke, *Dismembering the Male*, 33.

[17] For examples, see Glamorgan Archives, DPD/2/5/6/82, Powell Duffryn Company, Letters from Douglas A. Hann to hospitals, doctors, etc. to give orders for medical treatment for artificial limbs, knee splints, etc. for injured men, 1926; and DPD/2/5/6/206, Powell Duffryn Company, Applications for tickets for Porthcawl Rest, 1926.

[18] South Wales Miners' Federation (SWMF) Annual Conference minutes, 14–17 June 1920 (South Wales Coalfield Collection, Swansea University: SWCC/MNA/NUM/3/1/1/).

[19] *Tarian y Gweithiwr*, 2 May 1901.

[20] *Merthyr Telegraph*, 22 July 1865.

[21] Francis Hywel Smith David, The Fed: A History of the South Wales Miners in the Twentieth Century (London: Lawrence & Wishart, 1980).

[22] Jones, ‘Workmen's Compensation and the South Wales Miner’, 155.

[23] Foot Michael, Aneurin Bevan, A biography vol. 1 (London: MacGibbon and Kee, 1963), 63; David G. Green, *Working-Class Patients and the Medical Establishment* (Aldershot: Maurice Temple Smith Ltd., 1985), 174.

[24] In the south Wales coalfield, ‘lodge’ was the term used for a colliery work-place branch of the Miners' Federation. In this example, therefore, the Bute Merthyr Lodge was the union branch at the Bute Merthyr colliery. Copy of letter to J. M. Williams (Bute Merthyr Lodge) from SWMF Compensation Department, 25 September 1934. SWMF Compensation Secretary's Correspondence with Area No.4, 1934–41 (SWCC/MNA/NUM/3/5/15).

[25] In 1933–4 the Federation reorganised its entire structure, replacing its nineteen constituent districts with eight larger and more efficient ‘areas’. *The Colliery Workers' Magazine*, October 1923, 1, 251; Maesteg District SWMF: Rules of Artificial Limb Fund, 1928 (SWCC/MNA/NUM/3/8/7); SWMF NUM (South Wales) Area No. 2 minutes, 1934–52 (SWCC/MNA/NUM/3/8/17(a)).

[26] SWMF Rhondda No. 1 District, Minutes of District Committee meeting held on 3 October 1921.

[27] Bourke, *Dismembering the Male*, 48.

[28] Maesteg District SWMF Artificial Limb Fund Rules.

[29] SWMF Area No. 2 minutes, 16 March 1936; 13 December 1943.

[30] The ‘poundage’ system was unique to south Wales and in use in medical schemes throughout the region. It consisted of weekly payments of a number of pennies, usually 2d. or 3d., per pound of income and was thus a basic form of income tax whereby better-paid workers helped to subsidise the costs of the medical care of less well-paid workers and their families. As such, it was a more progressive form of funding than the more regressive system of flat-rate contributions in place in medical schemes in other parts of Britain.

[31] Tredegar Workmen's Medical Aid Society (TWMAS), General Committee minutes, 29 January 1924 (Gwent Archives, D.3246.1).

[32] TWMAS, Special General Meeting, 30 July 1928.

[33] Ebbw Vale Workmen's Medical Society (EVWMS) Committee Minute Book, Monthly Hospital meetings, 16 March, 18 May 1916; 16 August 1917 (Gwent Archives, D.2472.10).

[34] Ebbw Vale Workmen's Doctors' Fund (EVWDF), minutes of monthly meeting, 31 December 1898 (Gwent Archives, D.2742.9).

[35] EVWMS Committee Minute Book, Monthly Hospital meeting, 17 November 1921.

[36] SWMF Area No. 2 minutes, 30 July 1934.

[37] SWMF Area No. 2 minutes, 16 November 1942.

[38] TWMAS, General Committee minutes, 17 September 1936.

[39] TWMAS, General Committee minutes, 9 June 1938.

[40] TWMAS, General Committee minutes, Special General Committee meeting minutes, 27 November 1933; General Committee minutes, 19 April 1934; 22 August 1935.

[41] TWMAS, General Committee minutes, 17 March 1938.

[42] EVWMS Committee Minute Book, Hospital Committee meeting, 20 March 1943.

[43] EDWDF Committee minutes, 17 November 1900; 7 May, 16 July 1904.

[44] TWMAS, General Committee minutes, 20 January 1938,

[45] SWMF Area No. 2 minutes, 6 March 1944.

[46] National Union of Mineworkers (South Wales) Area No. 2 minutes, 5 February 1945. The SWMF became the South Wales Area of the National Union of Mineworkers (NUM) in January 1945.

[47] This is very much the focus of Bourke, *Dismembering the Male*, for example.

[48] Artificial teeth were to be supplied only in the event of an industrial injury of sufficient extent as to destroy the whole of the top or bottom set of natural teeth, or the whole top or bottom set of existing artificial teeth. Maesteg District SWMF Artificial Limb Fund Rules.

[49] TWMAS, General Committee minutes, 17 June, 24 September 1912; 29 January 1924.

[50] EVWDF, minutes of monthly meeting, 27 August 1898.

[51] TWMAS, General Committee minutes, 14 February 1928.

[52] EVWMS, Hospital Committee minutes, 19 February 1944.

[53] Truss and Wooden Leg Register, Dowlais Iron Company Employees, 1891–1902. Cresswell Family Practice Records, Dowlais (Glamorgan Archives, DX83/9/1).

[54] A spinal carriage was a type of ‘wheelchair’ in which a person lay horizontally, rather than sat upright. It was used by individuals whose spinal injuries meant that they were unable to be transported by any other means.

[55] TWMAS, General Committee minutes, 31 July 1923; 18 May, 1, 15, 22 June, 13, 27 July, 10 August 1933; 17 May, 12 July 1934; 8 August 1935; 11, 22 June, 9 July, 3 September 1936; General Purpose Committee minutes, 16 August 1923; 13 March 1924.

[56] TWMAS Finance Committee minutes, 14 December 1922.

[57] TWMAS, General Committee minutes, 26 July 1912; 18 May 1933; 28 May 1936; General Purpose Committee minutes, 13 September 1921.

[58] SWMF/South Wales NUM Area No. 2 minutes, 18 December 1939; 13 December 1948.

[59] For instance, see SWMF/South Wales NUM Area No. 2 minutes, passim.

[60] South Wales NUM Area No. 2 minutes, 17 September 1945.

[61] Bourke, *Dismembering the Male*, 45.

[62] TWMAS, General Committee minutes, 19 April 1934.

[63] This figure includes all surgical appliances, not just artificial limbs. It has been calculated from information contained in TWMAS Annual Balance Sheets, Statements of Accounts and Reports, 1916–48 (Gwent Archives, D3246.16.2–32). The accounts for 1925 and 1926 are absent.

[64] SWMF Area No. 2 Rules for Artificial Limb Fund. These Rules appear in the Area No. 2 minutes, inserted in between the meetings held on 13 December 1943 and 10 January 1944.

[65] TWMAS, General Committee minutes, 30 October, 15, 28 November 1935; 30 April, 14, 28 May, 17 September 1936.

[66] TWMAS, General Committee minutes, 20 January 1938.

[67] ‘A Window on Wales: 1912–31’, National Eisteddfod of Wales website, <http://www.eisteddfod.org.uk/english/content.php?nID=839>, accessed 17 April 2013.

[68] TWMAS, General Committee minutes, 14 October 1922; Finance Committee minutes, 10 November 1922; 30 May 1923.

[69] For a more detailed technical discussion of this subject, see Guyatt, ‘Better Legs’, 307–25.

[70] Bourke, *Dismembering the Male*, 47.

[71] EVWDF, minutes of monthly meeting, 27 March 1897.

[72] Guyatt, ‘Better Legs’, 307, 315–21.

[73] TWMAS, General Committee minutes, 19 April 1934; 30 April, 11 June, 17 September, 23 December 1936; 24 June, 13, 27 September 1937.

[74] Guyatt, ‘Better Legs’, 316, 321; Bourke, *Dismembering the Male*, 46.

[75] Bourke, *Dismembering the Male*, 47.

[76] EVWDF, Special meeting minutes, 3 February, 3 April 1906.

[77] EVWMS, Monthly Hospital meeting, 20 September 1917.

[78] TWMAS, Finance Committee minutes, 14 May 1921; General Committee minutes, 29 April, 1 June 1921; General Purpose Committee, 21 July 1921.

[79] Anderson, *War, Disability and Rehabilitation*, 25.

[80] TWMAS, General Committee minutes, 24 June, 25 November 1937; 9 June 1938.

[81] TWMAS, General Committee minutes, 20 January 1938.

[82] TWMAS, General Committee minutes, 25 November, 9 December 1937; 17, 31 March, 14 May, 4 July, 4 August 1938.

[83] SWMF Area No. 2 minutes, 16 March 1936.

[84] TWMAS, General Committee minutes, 15 March 1927; 21 June 1928; 8 May 1930; 9 July 1931; 30 May, 28 June, 22 August 1935; 20 February 1936.

[85] TWMAS, General Committee minutes, 9 June 1938.

[86] Maesteg District SWMF Artificial Limb Fund Rules; EVWMS, Monthly meeting, 26 June 1915; Monthly Hospital meeting, 18 January, 17 May 1917; 17 June 1920.

[87] TWMAS, General Committee minutes, 30 October, 15, 28 November 1935.

[88] TWMAS, General Committee minutes, 30 April, 14 May, 11 June, 17 September 1936; 31 March 1938.

[89] TWMAS, General Committee minutes, 17 September 1936. He also appears in the minutes on several occasions prior to this regarding his artificial leg—see entries for 28 February 1929; 9, 23 March 1933; 28 November 1935.

[90] TWMAS, Annual Meeting for the Appointment of Representatives, 4 April 1936, General Committee minutes, 30 April, 14 May, 11 June, 17 September 1936; 31 March 1938. It was common in south Wales, throughout the twentieth century, for miners disabled from working to become union lodge officials; this was a further means of assistance offered by the union to disabled members.

[91] TWMAS, General Committee minutes, 23 October 1939.

[92] Letter to Evan Williams (SWMF Compensation Secretary) from J. M. Williams (Bute Merthyr Lodge), 21 September 1934. SWMF Compensation Secretary's Correspondence with Area No. 4, 1934–41.

[93] Dowlais SWMF applications for compensation, 1939–46 (S.O. Davies Papers, Glamorgan Archives, DXHV/37); Collieries and iron works medical reports on injured workmen, 1914–16 (Dowlais Iron Company Collection, Glamorgan Archives, DG/11/1).

[94] Williams-Searle, ‘Cold Charity’, 157–86.

